# Hepatokines lipocalin 2 and osteopontin drive muscle atrophy in MASH^☆^

**DOI:** 10.1016/j.molmet.2026.102391

**Published:** 2026-06-10

**Authors:** Amy R. Fumo, Simon I. Dreher, Pauline Morigny, Honglei Ji, Raul Terron Exposito, Tuna F. Samanci, Tushar More, Lara Ruoff, Joël J. Tissink, Carmen Paredes Yubero, Ana Jimena Alfaro, Christine von Toerne, Stefanie M. Hauck, Sophie H.A. Nusser, Zoltan Czigany, Kenneth A. Dyar, Stephan Herzig, Mauricio Berriel Diaz, Anja Zeigerer, Karsten Hiller, Theresa H. Wirtz, Cora Weigert, Maria Rohm

**Affiliations:** 1 Institute for Diabetes and Cancer (IDC), Helmholtz Munich, Neuherberg, Germany; 2 Joint Heidelberg-IDC Translational Diabetes Program, University Hospital, Heidelberg, Germany; 3 German Center for Diabetes Research (DZD), Munich, Germany; 4 Institute for Clinical Chemistry and Pathobiochemistry, Department for Diagnostic Laboratory Medicine, University Hospital Tübingen, Tübingen, Germany; 5 Institute for Diabetes Research and Metabolic Diseases (IDM) of Helmholtz Munich at the University of Tübingen, Tübingen, Germany; 6 Department of Biochemistry and Bioinformatics, Technische Universität Braunschweig, Braunschweig, Germany; 7 European Center for Angioscience, Medical Faculty Mannheim, Heidelberg University, Mannheim, Germany; 8 Metabolomics and Proteomics Core Facility, Helmholtz Munich, Neuherberg, Germany; 9 Medical Department III, University Hospital RWTH Aachen, Aachen, Germany; 10 Department of General-, Visceral- and Transplantation Surgery, Heidelberg University Hospital, Heidelberg, Germany; 11 German Center for Cardiovascular Research (DZHK), Munich, Germany

## Abstract

A bidirectional relationship exists between metabolic dysfunction-associated steatotic liver disease (MASLD) and its progressive inflammatory form, metabolic dysfunction-associated steatohepatitis (MASH), and sarcopenia, with each worsening the prevalence and prognosis of the other. Hepatokines have recently been shown to affect skeletal muscle metabolism and function, both in the context of MASLD and wasting diseases. We here explored the possibility of targeting hepatokines to counteract MASLD-induced sarcopenia. Integrating mouse and human liver transcriptomics with muscle proteomics from MCD- and GAN-diet induced murine MASH models with sarcopenia, we identified three MASH-induced hepatokines, namely LCN2, LGALS3 and OPN. These hepatokines were elevated in the circulation of mouse MASH models with sarcopenia and in sarcopenic patients with advanced chronic liver disease. C2C12 myotubes treated with liver-secreted proteins as well as recombinant LCN2 and LGALS3 exhibited atrophy. Stable isotope tracing and mitochondrial respiration showed that liver-secreted proteins altered mitochondrial metabolism in C2C12 myotubes, which was recapitulated in primary human myotubes. Human 3D skeletal muscle organoids treated with recombinant proteins exhibited functional impairment. Virus-mediated knockdown of LCN2 in liver of mice with MASH improved muscle function and myotube size, whereas virus-mediated overexpression of LCN2 in the liver aggravated MASH-induced myotube atrophy. Targeting hepatokines may therefore be a feasible future therapeutic strategy against sarcopenia.

## Highlights

• Mouse models with MASH displayed sarcopenia and muscle mitochondrial dysfunction.
• Integration of multiple -omics datasets identified MASH-induced hepatokines.
• Liver secreted proteins induced myotube atrophy and metabolic/functional impairment.
• Liver specific targeting in MASH mice improved sarcopenia.
• Patients with liver disease & sarcopenia had high circulating lipocalin2/osteopontin.

## Graphical abstract

**Figure:**
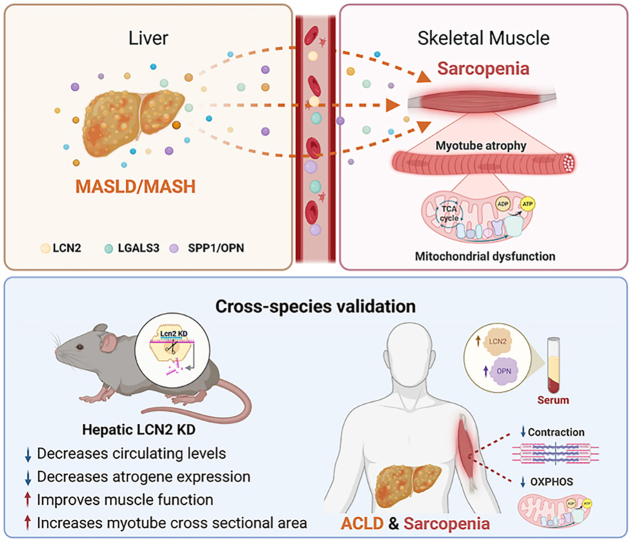

## 1 Introduction

Metabolic dysfunction-associated steatotic liver disease (MASLD) [1] and its progressive inflammatory form, metabolic dysfunction-associated steatohepatitis (MASH), are now leading causes of chronic liver disease and major drivers of cardiometabolic morbidity and mortality. MASLD affects an estimated third of the global population, with higher prevalence in men than in women [2], and is expected to increase further in upcoming years. MASLD spans simple steatosis to steatohepatitis with fibrosis, cirrhosis, and hepatocellular carcinoma, and is tightly linked to insulin resistance, systemic low-grade inflammation, and altered endocrine signaling. Within this framework, the liver is increasingly viewed as an endocrine organ, secreting hepatokines which exert metabolically relevant effects on distant tissues, including skeletal muscle [3,4].

Sarcopenia, defined by progressive loss of skeletal muscle mass and strength, is highly prevalent in individuals with metabolic disease and independently predicts frailty, disability, and mortality. It is also particularly frequent in patients with MASLD/MASH, and clinically relevant: meta-analyses show a higher prevalence of sarcopenia in patients with MASLD compared with controls, and sarcopenia is associated with a poor prognosis in these patients [5]. Although the reported prevalence rates of sarcopenia in MASLD vary widely due to heterogeneity in both sarcopenia assessment methods and MASLD diagnostic criteria, numerous studies have established a correlation between muscle wasting and MASLD. A recent systematic review analyzing data from 24 cross-sectional studies found that patients with MASLD have a 74% increased risk of developing sarcopenia [6]. Furthermore, sarcopenia has been associated with an increased risk of MASLD progressing to advanced fibrosis and MASH, as well as higher mortality rates, insulin resistance, and cardiovascular events [7].

Clinical and experimental evidence supports a bidirectional liver-muscle axis in MASLD/MASH, in which hepatic and muscular pathologies amplify one another. MASLD-related insulin resistance, chronic inflammation, and altered hepatokine secretion promote muscle protein breakdown, impaired regeneration, and myosteatosis, thereby accelerating sarcopenia, while sarcopenia and muscle insulin resistance aggravate systemic metabolic stress and hepatic steatosis, fostering progression to MASH and fibrosis [3]. Patients with coexisting MASLD/MASH and sarcopenia consequently exhibit poorer outcomes than those with either condition alone [8].

Key mechanistic work has begun to dissect hepatokine-mediated liver-muscle crosstalk in MASLD. Quantitative proteomics of hepatocytes isolated from healthy and steatotic mouse livers has demonstrated that the hepatocyte secretome undergoes significant changes in response to liver steatosis [9]. Hepatokines secreted from livers of mice with MASLD have been shown to cause myotube insulin resistance and protein breakdown [10], fostering the idea that liver-secreted factors specific for MASLD directly contribute to muscle functional impairment. This is exemplified by the described role of liver-secreted Fetuin-A, circulating levels of which are consistently elevated in MASLD [11], and which directly acts on the muscle to impair insulin signaling [12]. Overall, these studies highlight the bidirectional relationship between MASLD/MASH and sarcopenia and the involvement of hepatokines therein, whereas unbiased approaches aiming at identifying responsible hepatokines are still rare.

Here, we employ two MASH mouse models to describe the interaction between MASH and sarcopenia, and integrate proteomics data from these mice with published human and mouse liver transcriptomics datasets to identify liver-secreted factors contributing to muscle wasting in an unbiased approach. We demonstrate that the identified hepatokines cause myotube atrophy and metabolic alterations, and their circulating levels in patients with advanced chronic liver disease are associated with sarcopenia. Lastly, we demonstrate with the example of lipocalin 2, that inhibiting liver-derived secretion reduces sarcopenia, while overexpression in liver is sufficient to induce myotube atrophy. Overall, our study supports the concept that MASLD/MASH-specific hepatokines such as lipocalin 2 constitute key effectors of liver-muscle crosstalk and promising therapeutic targets to counteract sarcopenia.

## 2 Results

### 2.1 MASH is associated with muscle atrophy in MCD and GAN mice

**Figure 1:**
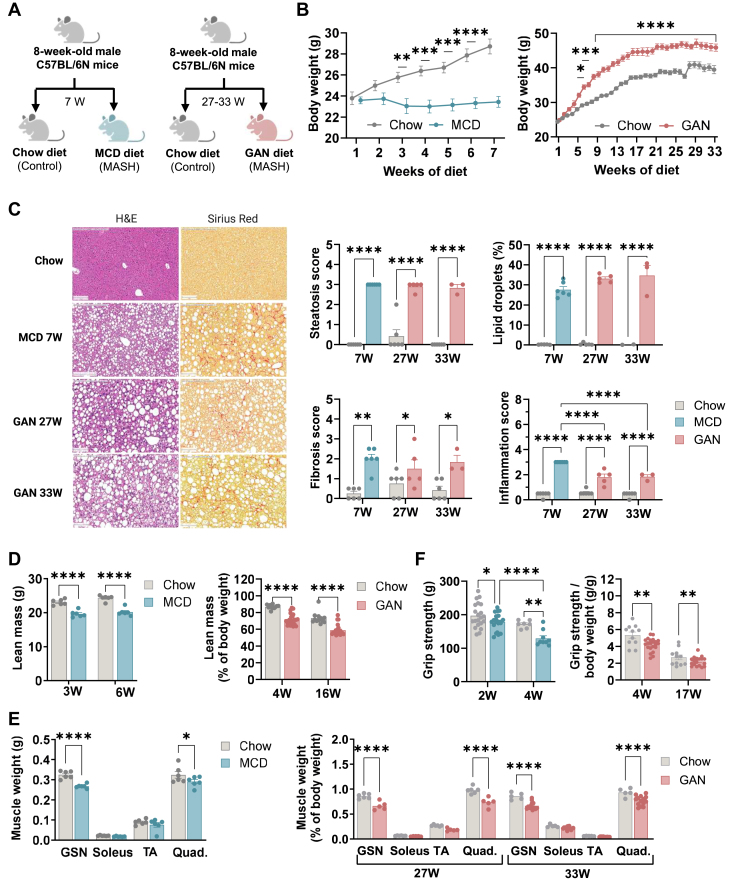
**Metabolic Dysfunction-Associated Steatohepatitis (MASH) is associated with muscle atrophy in MCD and GAN mice. (a)** Experimental design of MCD (7W) and GAN (27–33W) feeding protocols in male C57BL/6N mice. (**b)** Body weight progression in MCD and GAN-fed mice. **(c)** Representative H&E and Sirius Red stained liver sections with quantification of steatosis, lipid droplets, fibrosis, and inflammation. MCD n = 6 per group; GAN 27W n = 5, chow 27W n = 6; GAN 33W n = 3, chow 33W n = 6. **(d)** Lean mass determined by EchoMRI in MCD (3W, 6W; n = 6 per group) and GAN-fed mice (4W, 16W: chow n = 12, GAN n = 24 (4W) and n = 22 (16W)). **(e)** Muscle weights of gastrocnemius (GSN), soleus, tibialis anterior (TA), and quadriceps (Quad.) in MCD at 7W (n = 6 per group), and GAN mice at 27W (GAN n = 5, chow n = 6) and 33W (GAN n = 15, chow n = 5). All tissues were collected at experimental endpoint. (**f)** Four limb grip strength (absolute and normalized). MCD: 2W n = 21 per group; 4W chow n = 7, MCD n = 10. GAN: 4W chow n = 12, GAN n = 22; 17W chow n = 12, GAN n = 18. Data are presented as mean ± SEM. Statistical analyses were performed using two-tailed Student's t-test or two-way ANOVA with Šídák's multiple comparison post hoc test. ∗P < 0.05, ∗∗P < 0.01, ∗∗∗P < 0.001, ∗∗∗∗P < 0.0001. Panel (a) created with BioRender.com.

With the aim to identify model-overarching associations between MASH and sarcopenia, we initially employed two independent mouse MASH models: the MCD model and the GAN model (Figure 1A). In the MCD model, mice were fed a methionine choline deficient high-fat (60% kcal) diet for 7 weeks. In the GAN model, mice were fed the Gubra Amylin NASH diet containing high-fat (40% kcal), high-fructose (22% kcal), and high-cholesterol (2%) for 27–33 weeks to mimic human MASLD. Control animals were fed a Chow diet.

While MCD mice had a lower body weight than respective controls, GAN mice gained body weight and fat mass in comparison to age-matched control animals, highlighting that the two models have different disease trajectories, similar to patients with MASH where obesity is not always present (Figure 1B,Supplemental Fig. 1a). Despite this difference in body weight development, MASH scores progressed similarly, with MCD and GAN animals displaying significant accumulation of lipids, and high fibrosis and inflammation scores (Fig. 1C). Elevated levels of circulating aspartate aminotransferase (AST) and alanine aminotransferase (ALT) confirmed liver injury in both models (Supplemental Fig. 1b, c).

To address the connection between MASH and sarcopenia, we next assessed muscle mass and function in both models. MCD and GAN mice both had reduced lean mass and smaller skeletal muscles (gastrocnemius (GSN) and quadriceps) compared to their respective controls (Fig. 1d, e). This was reflected at the level of reduced cross-sectional area (CSA) of type IIX and IIB fibers in GSN of MCD and type I, IIA and IIB fibers in GSN of GAN mice (Supplemental Fig. 1d). Lipid accumulation was not significantly different in both MCD and GAN muscles (Supplemental Fig. 1e). A significant increase in the number of IIB fibers, accompanied by a tendency towards a decrease in the percentage of IIX fibers, in GSN muscle of MCD mice suggested a shift toward a more glycolytic phenotype in this model (Supplemental Fig. 1f). Individual fiber types are therefore differentially affected by atrophy between different models. Both MCD and GAN muscles had a higher percentage of small to medium sized muscle fibers (1000–2000 μm), and MCD muscles had a lower percentage of large fibers (3000–4000 μm) (Supplemental Fig. 1f, g). In line with sarcopenia-associated functional impairment, both MCD and GAN mice had reduced grip strength (Fig. 1F).

In summary, both MCD and GAN mice displayed features of MASH and associated sarcopenia.

### 2.2 Proteomic analysis of muscles from MASH mice reveals mitochondrial function as commonly dysregulated pathway

**Figure 2:**
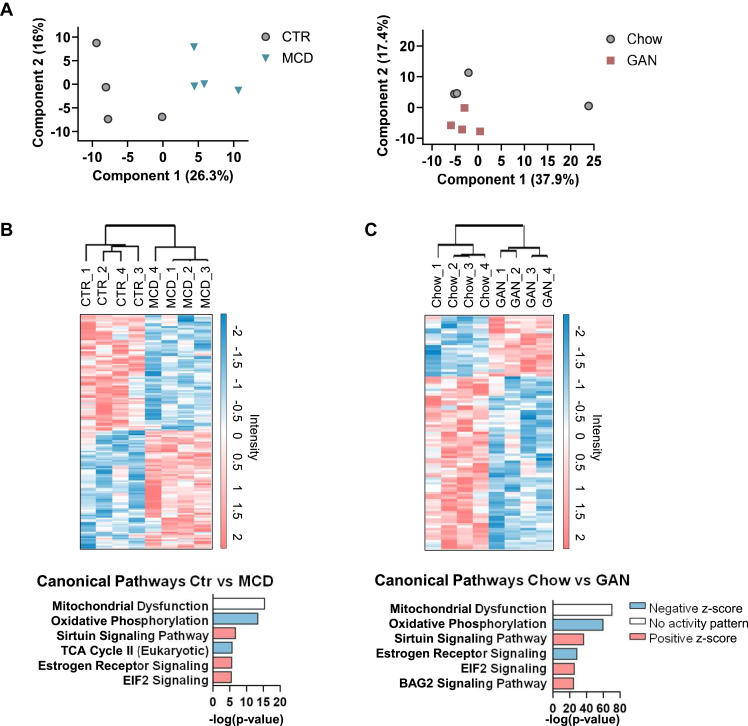
**Proteomic profiling of skeletal muscle identifies shared pathway alterations across MASH. (a)** Principal component analysis (PCA) of gastrocnemius (GSN) muscle proteomes from control and MCD-fed mice (n = 4 per group) and chow- and GAN-fed mice (27W). **(b)** Hierarchical clustering heatmap of differentially expressed proteins in GSN muscle from MCD vs. Ctr. Rows represent proteins and columns individual samples. Protein abundance is shown as Z-score-normalized label-free quantitative (LFQ) proteomics intensities (red, upregulated; blue, downregulated). Only proteins with adjusted p < 0.05 were included. Top enriched canonical pathways identified by Ingenuity Pathway Analysis (IPA), based on significantly altered proteins (adjusted p < 0.05), are shown alongside the heatmap. **(c)** Corresponding heatmap and pathway analysis for the GAN model (27W) compared to its respective control.

To better characterize the molecular signatures of MASH-induced sarcopenia, we next performed a proteomics analysis of GSN muscle of both MCD and GAN mice (Figure 2). The muscle proteomes varied substantially between control and MASH animals, with 200 and 97 proteins altered in MCD and GAN muscles, respectively (adj p < 0.05) (Fig. 2A). Hierarchical clustering showed that muscles of animals with MASH clustered together (Fig. 2b, c). Pathway analysis of proteins altered between control and MASH animals revealed prominent changes in mitochondrial pathways, including “mitochondrial dysfunction” and “oxidative phosphorylation” (Fig. 2b, c). These pathways were consistently regulated in the same direction in both MCD and GAN mice. Of note, while some proteins were consistently regulated in both models, some were unique to the individual condition, yet part of the same pathways (Supplemental Table 1, 2). Among mitochondrial proteins, VDAC2 was significantly altered in both MCD and GAN muscles (-log10 p = 1.475 and 1.855, respectively). Distinct alterations in antioxidant-associated proteins were also observed, with PRDX5 significantly altered in MCD muscles (-log10 p = 1.878) and PRDX3 in GAN (-log10 p = 1.833). Additional mitochondrial proteins selectively altered in MCD muscles included SDHA (-log10 p = 1.541) and VDAC3 (-log10 p = 1.528), whereas GAN muscles showed altered expression of the mitochondrial fission protein FIS1 (-log10 p = 1.614).

### 2.3 Liver-secreted factors induce myotube atrophy and alter myotube mitochondrial function and metabolism

**Figure 3:**
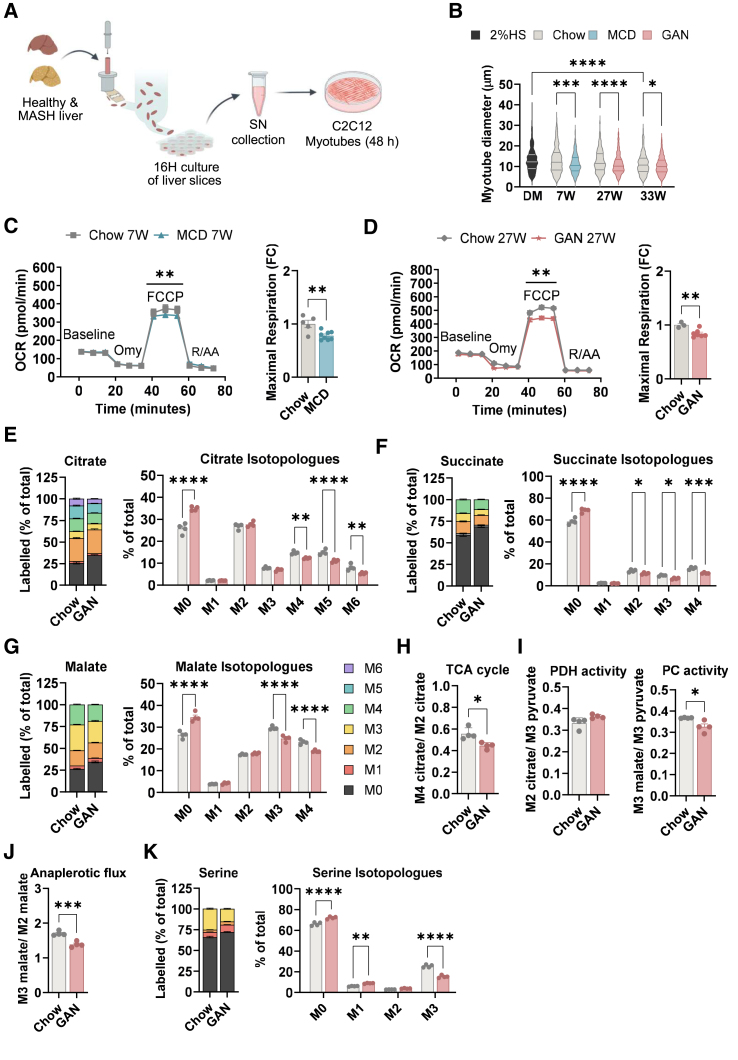
**Liver-derived factors from MASH mice induce myotube atrophy and impair mitochondrial respiration and TCA cycle flux. (a)** Schematic of the precision-cut liver slice workflow used to generate liver-derived supernatant (SN) for myotube atrophy and functional assays. **(b)** C2C12 myotube diameter (μm) following treatment with concentrated liver-derived SN (3 kDa cutoff) from chow or MCD mice (n = 3; ed analysis). **(c)** Oxygen consumption rate (OCR) measured by Seahorse XF Mito Stress Test in C2C12 myotubes following 48 h treatment with SN from chow (n = 5) or MCD (n = 8) mice. Representative OCR traces and maximal respiration quantification are shown. **(d)** OCR measurements and maximal respiration in C2C12 myotubes treated with SN from chow (n = 3) or GAN (n = 7) mice. **(e**–**g)** Isotopic tracing analysis of tricarboxylic acid (TCA) cycle intermediates in C2C12 myotubes following 48 h incubation with SN fom 27-week-fed chow or GAN mice. Total labeled metabolite fractions and corresponding isotopologue distributions are shown for citrate **(e)**, succinate **(f)**, fumarate **(g)** (n = 4). **(h**–**j)** Ratios derived from isotopic tracing reflecting TCA cycle activity (M4 citrate/M2 citrate), pyruvate dehydrogenase (PDH) activity (M2 citrate/M3 pyruvate), pyruvate carboxylase (PC) activity (M3 malate/M3 pyruvate), and anaplerotic flux (M3 malate/M2 malate). **(k)** Isotopic tracing analysis of serine after treatment as in (e–g). Statistical analyses were performed using two-tailed Student's t-test or one-/two-way ANOVA with Šídák's multiple comparison post hoc test. ∗P < 0.05, ∗∗P < 0.01, ∗∗∗P < 0.001, ∗∗∗∗P < 0.0001.

To assess if liver-secreted factors were able to directly induce myotube atrophy and alter muscle mitochondrial function, we isolated livers of MASH mice and generated precision cut liver slices by preparing uniform tissue sections and culturing them *ex vivo* under oxygenated conditions, thereby preserving native hepatic architecture and cellular composition to assess liver-derived secreted factors (Figure 3A). We assessed the effect of liver-derived factors on myotubes by treating C2C12 myotubes with supernatant (SN) collected from live liver slices. Concentrated (>3 kDa) SN from both MCD and GAN MASH mice induced atrophy in C2C12 myotubes (Fig. 3B), confirming that liver-derived proteins directly induced myotube wasting.

As mitochondrial dysfunction was the top pathway altered in the MASH-associated muscle proteome in both MCD and GAN mice, we next assessed the effect of MASH on mitochondrial function in myotubes. We treated C2C12 myotubes with SN from live liver slices and assessed mitochondrial function using both Seahorse extracellular flux analysis and [U–^13^C]-glucose tracing. SN from both MCD and GAN liver slices induced impaired maximal mitochondrial respiration, in line with mitochondrial dysfunction in these atrophic conditions (Fig. 3c, d). Stable isotope tracing revealed increased levels of unlabeled metabolites of the tricarboxylic acid cycle (TCA) in myotubes treated with GAN SN, specifically citrate, succinate, and malate, while labelled metabolites (M3 and higher) were significantly reduced in MASH vs. control SN treated myotubes (Figure 3e–G). This indicated a reduced entry of glycolytic carbon into the TCA cycle, coupled with impaired cycling activity. Indeed, TCA cycle activity (approximated by M4 citrate/M2 citrate) was significantly reduced in atrophic myotubes (Fig. 3H). This may be partially caused by a reduced activity of anaplerotic pyruvate carboxylase (PC), as PC but not pyruvate dehydrogenase (PDH) activity levels were significantly reduced in atrophic myotubes (Fig. 3I), and anaplerotic flux (approximated by M3/M2 malate) was decreased (Fig. 3J). Overall, tracing data confirmed impaired mitochondrial metabolism of atrophic myotubes treated with SN of MASH livers, compared to myotubes treated with SN of control livers.

In addition, glucose tracing revealed alterations in serine/glycine one-carbon metabolism in atrophic myotubes treated with SN from GAN animals (Figure 3K, Supplemental Fig. 2a). Consistent with reduced *de novo* serine biosynthesis from glucose, the enrichment of serine M3 derived from [U–^13^C]glucose was significantly decreased in GAN compared with Chow, while glycine M2 labeling was only minimally increased, suggesting that glycine labeling from glucose was maintained through compensatory network activity. In parallel, GAN SN treated myotubes exhibited increased serine M1 and M2 isotopologues, indicating enhanced isotopic exchange within the serine/glycine one-carbon network. These findings suggest that GAN induces a reorganization of serine/glycine metabolism characterized by reduced *de novo* serine production from glycolytic intermediates but increased reliance on reversible serine hydroxymethyltransferase -mediated one-carbon cycling. Pyruvate and lactate labelling from [U–^13^C]glucose were slightly reduced, indicating mildly impaired glycolysis (Supplemental Fig. 2b, c).

In summary, proteins secreted from live liver slices of MASH livers induced metabolic alterations possibly contributing to myotube atrophy.

### 2.4 Proteome analysis of liver-derived supernatant identifies candidate hepatokines inducing muscle wasting

**Figure 4:**
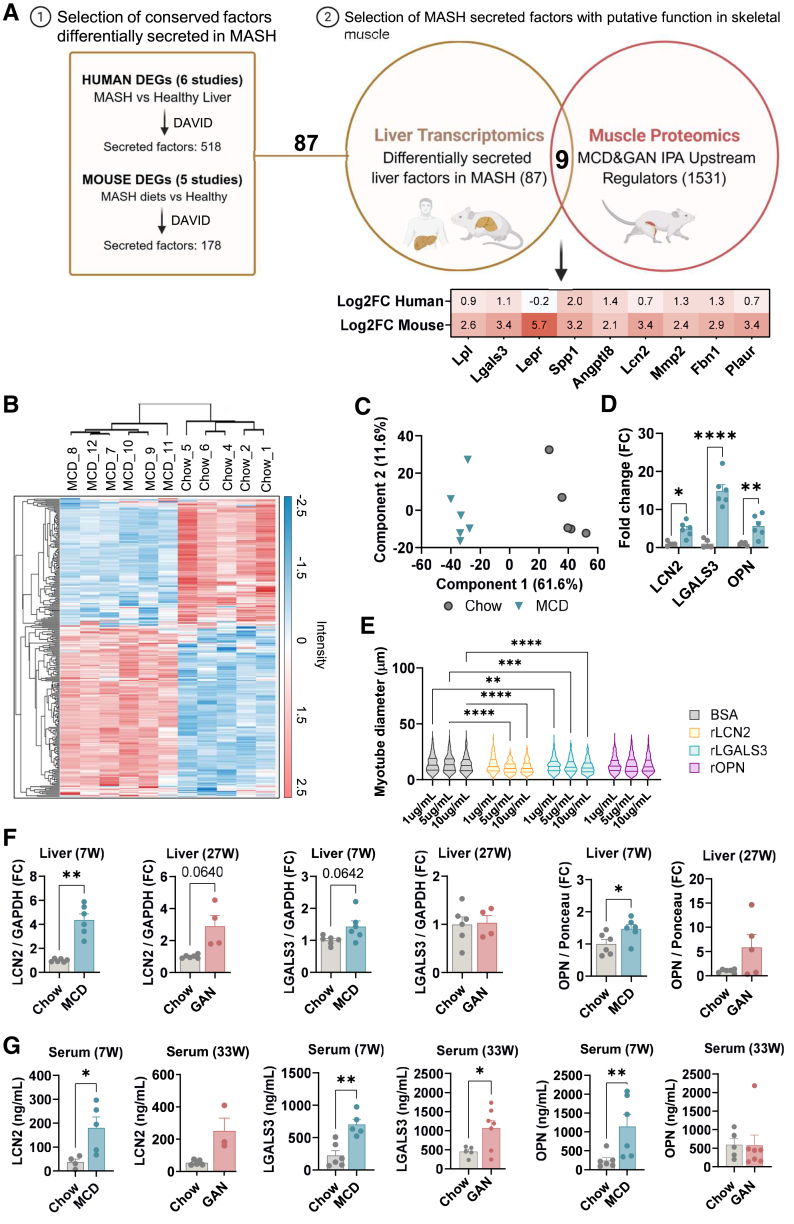
**Proteome analysis of liver-derived supernatant identifies candidate hepatokines inducing muscle wasting. (a)** Integrative workflow for the identification of liver-secreted candidate factors. Publicly available human (n = 6) and murine (n = 5) liver transcriptomic datasets were analyzed using Genevestigator®. Differentially expressed genes (adjusted p < 0.05; murine datasets additionally filtered for log2FC > 2) were screened for secreted proteins using DAVID functional annotation. Overlap of differentially secreted liver factors (87 common candidates) with predicted upstream regulators identified from muscle proteomics (IPA; 1,531 regulators) yielded 9 shared candidate mediators. These candidates are displayed in a heatmap showing the mean Log_2_ fold change (Log_2_FC) across the analyzed mouse and human studies. **(b)** Secretome proteomics of liver-derived SN from chow (n = 5) and MCD (n = 6) mice. Hierarchical clustering heatmap of differentially abundant proteins. Rows represent proteins and columns individual samples. Normalized protein abundance is shown (red: upregulated; blue: downregulated). Only proteins with adjusted p < 0.05 were included. **(c)** Principal component analysis (PCA) of liver-derived secretome proteomics. **(d)** Fold change of LCN2, LGALS3, and OPN in liver-derived secretome from MCD compared to chow mice. **(e)** C2C12 myotube diameter following treatment with recombinant LCN2, LGALS3, or OPN (n = 3, >500 myotubes, blinded). **(f)** Hepatic protein expression of LCN2, LGALS3 and OPN in MCD (n = 6/group) and GAN (27W: chow n = 6, GAN n = 4–5) models. **(g)** Circulating levels of LCN2, LGALS3 and OPN measured by ELISA. MCD model: chow n = 6, MCD n = 5–6 (LCN2: chow n = 4, MCD n = 5). GAN model (33W): chow n = 5, GAN n = 7 (LCN2: chow n = 5, GAN n = 3). Data are presented as mean ± SEM. Statistical analyses for quantitative data were performed using two-tailed Student's t-test or one-/two-way ANOVA with Šídák's multiple comparison post hoc test. ∗P < 0.05, ∗∗P < 0.01, ∗∗∗P < 0.001, ∗∗∗∗P < 0.0001. Panel (a) created with BioRender.com.

In order to identify liver-secreted factors driving sarcopenia in MASH, we next integrated our muscle proteomics data with published liver transcriptomics data (Figure 4A). Differentially expressed genes (DEGs) from six human and five murine studies were analyzed using the Genevestigator® platform [[13], [14], [15], [16], [17], [18], [19], [20], [21], [22]]. The identified DEGs were analyzed with the DAVID bioinformatics database to identify secreted factors. This analysis yielded 518 human and 178 murine differentially expressed genes encoding proteins that were annotated as secreted, in at least one study, of which 87 were commonly altered liver-derived factors in MASH. Then, we performed an Upstream Regulator analysis of the muscle proteomes from both MCD and GAN mice using Ingenuity Pathway Analysis, and integrated the resulting 1,531 common upstream regulators with the 87 liver-secreted factors, yielding a list of 9 MASH-specific, liver-secreted factors influencing muscle proteomes in MCD and GAN mice (Fig. 4A). These factors were: Lipoprotein lipase (*Lpl*), galectin 3 (*Lgals3*), leptin receptor (*Lepr*), osteopontin (*Spp1*), angiopoietin like 8 (*Angptl8*), lipocalin 2 (*Lcn2*), matrix metalloprotease 2 (*Mmp2*), fibronectin 1 (*Fbn1*), and plasminogen activator, urokinase receptor (*Plaur*).

We next sought to verify that the *in silico*-identified factors were indeed expressed in and secreted by the liver in MASH, and induced sarcopenia. We performed label-free quantitative (LFQ) proteomics on SN of control and MCD mice, identifying 294 differentially secreted proteins (adj p < 0.05) (Supplemental Table 3). Control and MCD SN clearly clustered apart in a principal component analysis, and hierarchical clustering further confirmed that the liver secretome differed between control and MASH (Fig. 4b, c). 6 out of our 9 candidate hepatokines were detected in the SN, and 3 were significantly enriched in MCD: LCN2, LGALS3, and osteopontin (OPN, encoded by *Spp1*) (Fig. 4D).

To assess their ability to induce atrophy, we treated C2C12 myotubes with increasing doses of recombinant proteins for 48 h, and found that rLGALS3 and rLCN2 - but not rOPN- dose-dependently induced C2C12 atrophy (Fig. 4E). This was accompanied by reduced expression of Eukaryotic translation initiation factor 4E-binding protein 1 (4E-BP1) upon rLCN2 treatment, and reduced phosphorylation of S6 ribosomal protein (S6RP) upon LCN2 and rOPN treatment, highlighting that these proteins directly impaired protein synthesis via the Mammalian target of rapamycin (mTOR) pathway (Supplemental Fig. 3a). Expression of mitochondrial complex proteins was reduced in C2C12 myotubes treated with rLCN2 and rLGALS3 (Supplemental Fig. 3b), consistent with impaired mitochondrial function of MASH-induced atrophic myotubes.

In conclusion, we identified MASH-induced hepatokines associated with sarcopenia and inducing myotube atrophy by affecting mitochondrial and protein synthesis pathways.

### 2.5 LCN2, LGALS3 and OPN are hepatocyte-secreted factors in MASH

**Figure 5:**
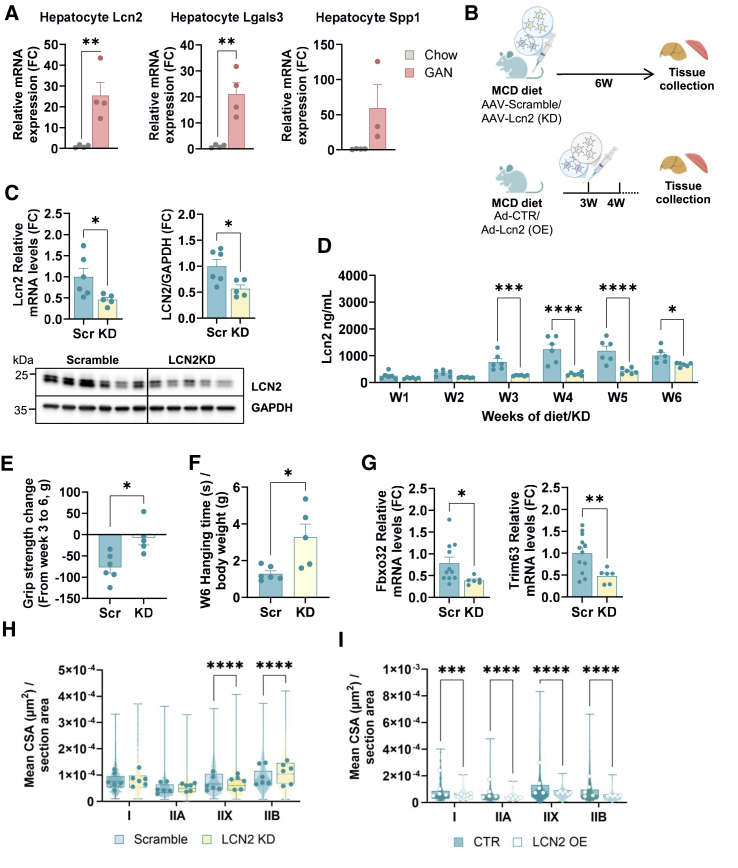
**Genetic alteration of hepatocyte *Lcn2* levels during MASH influences muscle function.(a)** Gene expression of *Lcn2, Lgals3, Spp1* (encoding OPN) in primary hepatocytes isolated from GAN- and chow-fed mice (27W; n = 4 per group; OPN GAN n = 3). **(b**) Schematic representation of hepatocyte-specific AAV-mediated knockdown (KD) and adenovirus-mediated overexpression (OE) of *Lcn2*. **(c)** Hepatic *Lcn2* expression following AAV-mediated knockdown in MCD-fed mice, compared to MCD-fed mice treated with scramble AAV (Scr), assessed by qPCR and LCN2 Western blot. Scr n = 6, *Lcn2* KD n = 5. **(d)** Serum LCN2 levels measured weekly by ELISA during the 6-week knockdown period (n = 6 per group). **(e)** Four-limb grip strength change from 3 to 6 weeks of MCD diet following *Lcn2* KD (n = 6 per group). **(f)** Four-limb hang test performance at 6 weeks of MCD diet following *Lcn2* KD (n = 6 per group). **(g)** GSN muscle gene expression of atrogenes *Fbxo32* and *Trim63* following *Lcn2* KD (*Lcn2* KD n = 6; MCD scramble n = 11). **(h)** Mean cross-sectional area (CSA) of skeletal muscle fiber types in *Lcn2* KD vs. Scr (n = 6 per group). CSA values were normalized to total section area. **(i)** Mean CSA of skeletal muscle fiber types in *Lcn2* OE vs. vector control mice (n = 6 per group). CSA values were normalized to total section area. Data are presented as mean ± SEM. Statistical analyses were performed using two-tailed Student's t-test or one-/two-way ANOVA with Šídák's multiple comparison post hoc test or uncorrected Fisher's LSD as appropriate. ∗P < 0.05, ∗∗P < 0.01, ∗∗∗P < 0.001, ∗∗∗∗P < 0.0001. Panel (b) created with BioRender.com.

To confirm that the proteins we identified in the supernatant of liver slices were also expressed in mouse livers, we measured their protein expression by Western Blot in livers of MCD and GAN mice and their respective controls. All 3 proteins were expressed in mouse livers, and LCN2 and OPN protein expression was increased in MASH models (p = 0.06 for LGALS3) (Figure 4F, Supplemental Fig. 4a, b). LCN2, LGALS3 and OPN protein levels were also detected in the circulation, and were elevated in the MCD MASH model and tended to be elevated in the circulation of the GAN MASH model (Fig. 4G), in line with their role as hepatokines. The gene expression of all three identified hepatokines was induced in isolated hepatocytes under MASH conditions (Figure 5A).

### 2.6 Genetic alteration of hepatocyte LCN2 levels during MASH influences muscle function

To causally link MASH-induced hepatokines with sarcopenia, we next modified their expression levels in hepatocytes to assess circulating levels and muscle size and function. We reduced hepatocyte LCN2 levels by treating animals with Adeno Associated Viruses (AAVs) encoding shRNAs targeting *Lcn2.* (Fig. 5B). Control animals received AAVs with a scrambled sequence. We confirmed knockdown efficiency by ∼60% and 40% on mRNA and protein levels, respectively (Fig. 5C), in mice fed a MCD diet. Hepatocyte-specific LCN2 knockdown significantly reduced circulating LCN2 levels throughout the course of the experiment (Fig. 5D), compared to the scramble control. LCN2 knockdown did not alter body weight, food consumption, liver pathology (AST, ALT), muscle weights, or fiber type composition of GSN muscles in MCD mice, compared to the scramble control (Supplemental Fig. 5a-f). Although total muscle sizes were unchanged, LCN2 knockdown significantly protected mice from MCD-induced impairments in muscle function, as assessed by grip strength and wire hang time (Figure 5e, F). Further, LCN2 knockdown reduced the mRNA expression of *Fbxo32* and *Trim63* (Fig. 5G), established atrogenes frequently used as molecular markers of muscle atrophy [23], [24], and caused significantly enlarged type IIB fibers (Fig. 5H), which represents the majority of fibers in GSN muscle. These data are in line with the notion that reducing LCN2 expression in and secretion from the liver in the MASH background reduced sarcopenia.

Next, we overexpressed LCN2 in hepatocytes by adenovirus-mediated expression of *Lcn2* (Fig. 5B). We confirmed that LCN2 was moderately overexpressed on mRNA and protein levels in liver and elevated in circulation, but was not expressed in muscle of mice under MCD diet (Supplemental Fig. 5g-i). LCN2 overexpression did not affect body weight, food consumption, muscle weights, or liver pathology in MCD mice, compared to the vector control (Supplemental Fig. 5j-m). However, hepatic LCN2 overexpression caused a reduced percentage of IIB fibers (Supplemental Fig. 5n), a shift from larger to smaller fibers (Supplemental Fig. 5o), and induced atrophy of all fiber types (types I, IIA, IIX, and IIB), evidenced by reduced cross-sectional area in GSN muscle (Fig. 5I).

Overall, genetic manipulation of liver LCN2 levels impacted muscle function and myotube sizes, in line with a direct role of liver-derived LCN2 in promoting muscle wasting in MASH.

### 2.7 Hepatokines induce mitochondrial dysfunction and functional impairment in human myotubes

**Figure 6:**
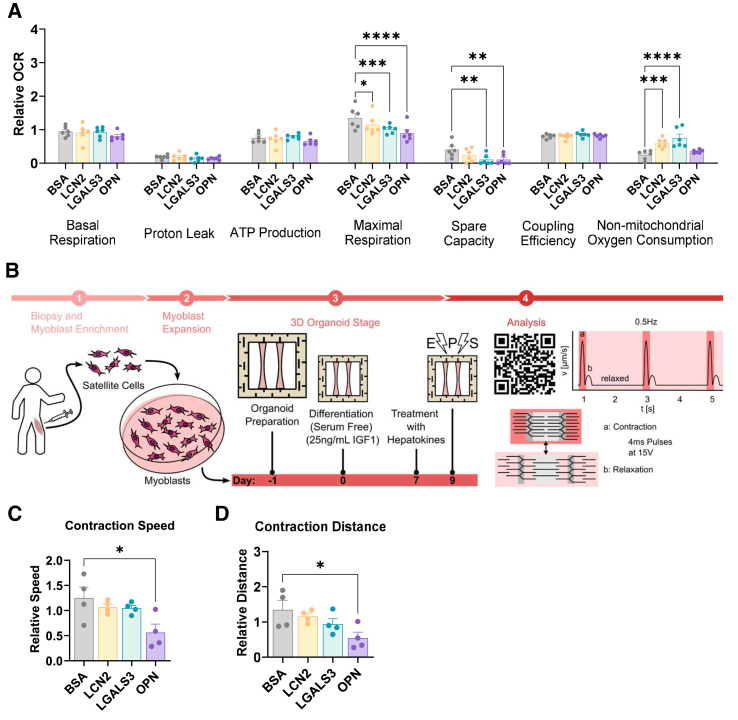
**Hepatokines cause mitochondrial and functional impairment in human myotubes and 3D skeletal muscle organoids. (a)** Mitochondrial respiration assessed by Seahorse XF Mito Stress Test (n = 6 independent donors) in functionally fully differentiated primary human myotubes treated with 1 μg/ml human recombinant LCN2, LGALS3, or 5 μg/ml OPN for 48h. Mitochondrial stages calculated from Seahorse parameters. **(b)** Schematic overview of 3D skeletal muscle organoid experiment. Primary human myoblasts isolated from vastus lateralis muscle biopsies were expanded, cast into 3D organoids with a physiological collagen based extracellular matrix anchored to a nylon frame and functionally differentiated for 7 days in the presence of 25 ng/ml IGF1. Organoids were treated with 1 μg/ml recombinant human LCN2, LGALS3 or 5 μg/ml OPN for 48 h before inducing controlled contraction at 0.5 Hz via electrical pulse stimulation (EPS). QR code leads to a representative sample video. Contraction **(c)** speed and **(d)** distance was analyzed in 15 s-videos captured after initiation of controlled contraction via EPS via a motion tracking algorithm [50]. Bar-plots show median and individual datapoints. n = 3–5 individual donors, mean ± SEM. Statistical analyses were performed using one-way ANOVA with Fisher's LSD or Bonferroni post hoc testing. ∗P < 0.05, ∗∗P < 0.01, ∗∗∗P < 0.001, ∗∗∗∗P < 0.0001.

To translate our findings to the human system, we treated functional primary human myotubes [25] with human recombinant LCN2, LGALS3 and OPN for 48 h before assessing mitochondrial respiration. Maximal respiration was reduced by all three hepatokines (Figure 6A). Spare capacity was reduced upon rLGALS3 and rOPN treatment, whereas non-mitochondrial oxygen consumption was increased upon rLCN2 and rLGALS3. These data support the overall negative implication by these MASH-derived hepatokines on mitochondrial respiration also in human myotubes. To assess hepatokines’ direct impact on human skeletal muscle functionality, we generated functional 3D human muscle organoids from myoblasts isolated from vastus lateralis biopsies. Fully differentiated organoids were treated with human recombinant LCN2, LGALS3 and OPN (Fig. 6B). While organoid diameter was unaffected by either hepatokine (Supplemental Fig. 6a), rOPN negatively impacted both speed and distance of organoid contraction initiated by electrical pulse stimulation (EPS) (Fig. 6c, d) Overall, this underlines that MASH-associated hepatokines alter mitochondrial function and muscle functionality also in human myotubes.

### 2.8 Circulating levels of atrophy-inducing hepatokines are increased in patients with ACLD and sarcopenia

**Figure 7:**
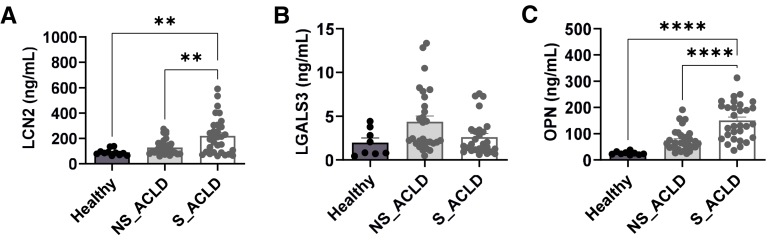
**Circulating levels of atrophy-inducing hepatokines are increased in patients with ACLD and sarcopenia.** Serum levels of LCN2 **(a)**, LGALS3 **(b)**, and OPN **(c)** measured by ELISA in male patients with advanced chronic liver disease (ACLD) with sarcopenia (n = 34), ACLD without sarcopenia (n = 35), and healthy controls (n = 11). Data are presented as mean ± SEM. Statistical analyses were performed using one-way ANOVA with Tukey's multiple comparison post hoc test. ∗∗P < 0.01, ∗∗∗∗P < 0.0001.

Lastly, to assess the relevance of our findings in humans, we measured circulating levels of LCN2, LGALS3 and OPN in serum from patients with advanced chronic liver disease (ACLD) and with or without sarcopenia. In these patients, sarcopenia was assessed by determining muscle area by CT scan at the height of the third lumbar vertebra, and defining the sex-specific skeletal muscle index (SMI; i.e. muscle area to square height of the patient). Clinical characteristics of patients are provided in Table 1. Grip strength for a subset of these patients was reported, and poor grip strength tended to correlate with low SMI (Supplemental Fig. 6b), in line with previous reports highlighting the utility of SMI in estimating functional performance [26]. LGALS3 was not significantly altered, whereas patients with ACLD and sarcopenia had significantly higher levels of circulating LCN2 and OPN compared to patients with ACLD without sarcopenia (Fig. 7a-c). This is in line with data from mice with MASH, and supports a role of these MASH-derived hepatokines in sarcopenia.

**Table 1:** **Clinical characteristics of male ACLD patients with and without sarcopenia and healthy controls.** Baseline demographic and clinical parameters of male patients with advanced chronic liver disease (ACLD) stratified by presence or absence of sarcopenia, and male healthy controls. Age, skeletal muscle index (SMI), body mass index (BMI), Model for End-Stage Liver Disease (MELD) score, and laboratory parameters are presented as mean (range). Sarcopenia was defined based on SMI criteria as described in the Methods. Abbreviations: ACLD, advanced chronic liver disease; SMI, skeletal muscle index; BMI, body mass index; MELD, Model for End-Stage Liver Disease; AST, aspartate aminotransferase; ALT, alanine aminotransferase; CRP, C-reactive protein.

	Sarcopenia	No Sarcopenia	
	ACLD	ACLD	Healthy
**n**	34	35	11
**Mean age [years] (Range)**	56.4 (21–75)	57.29 (27–69)	55.18 (52–61)
**Mean SMI [cm^2^/m^2^] (Range)**	34.83 (41.22–21.62)	56.96 (48.15–69.08)	
**Mean BMI [kg/m^2^] (Range)**	23.78 (15.06–41.87)	30.42 (21.26–43.77)	26.76 (21–32.70)
**Mean MELD (Range)**	20.68 (7–40)	16.63 (6–40)	
**AST [U/l] (Range)**	74 (14–231)	73.93 (16–183)	
**ALT [U/l] (Range)**	51.85 (10–177)	49.40 (10–192)	
**Albumin [U/l] (Range)**	3.16 (2.2–4.5)	3.37 (1.9–5)	
**Creatinine [mg/dl] (Range)**	1.88 (0.51–5.78)	1.22 (0.62–3.49)	
**CRP [mg/l] (Range)**	21.40 (0.7–96.5)	16.85 (0.6–170)

Overall, our data support the notion that LCN2 and OPN are hepatokines associated with MASH and sarcopenia in preclinical models and patients, relevant for myotube atrophy and functionality.

## 3 Discussion

In this study, we demonstrated that liver-derived proteins directly affected myotube size and function. Integrating mouse and human liver transcriptomics with muscle proteomics from murine MASH models with sarcopenia, we identified three hepatokines LCN2, LGALS3 and OPN, which were elevated in the circulation of mouse MASH models with sarcopenia and in sarcopenic patients with ACLD, and which induced changes to myotube size, function, and metabolism in mouse and primary human myotubes. By knocking down and overexpressing LCN2 in liver of mice with MASH, we demonstrated a direct connection between liver-derived LCN2 and muscle health, and provided proof-of-principle for targeting these circulating factors to counteract sarcopenia.

Sarcopenia is increasingly recognized as a frequent and clinically relevant extrahepatic manifestation of MASLD/MASH that worsens MASLD outcomes, and epidemiological data show that MASLD is an independent risk factor for reduced muscle mass and function, defining a bidirectional relationship [5,27]. This bidirectionality has recently been substantiated by Mendelian randomization and complementary mouse models showing that MASLD promotes muscle weakness, atrophy and ectopic lipid accumulation in skeletal muscle, while experimentally induced sarcopenia exacerbates liver steatosis, inflammation and fibrosis [28]. Mechanistically, our data demonstrated that atrophy under these conditions was associated with mitochondrial dysfunction and rewiring of metabolism. This is in line with the previously observed change in insulin sensitivity in myotubes treated with conditioned medium from livers of high fat diet fed mice [29]. Further, our finding that serine hydroxymethyltransferase -mediated serine -glycine/one-carbon cycling is increased aligns with the recent observation of increased one-carbon activity in myotubes of animals with cancer-induced atrophy [30], indicating that altered one-carbon metabolism may be a unifying feature of myotube atrophy.

The hepatic expression of LCN2, LGALS3 and OPN is robustly up-regulated in mouse and human MASLD/MASH liver [[13], [14], [15], [16], [17], [18], [19], [20], [21], [22]]. While LGALS3 directly induced changes to mitochondrial metabolism and myotube atrophy in our study, and has previously been associated with skeletal muscle remodelling and regeneration [31], we did not observe an association with ACLD-induced sarcopenia in patients.

In contrast, both LCN2 and OPN were strongly enriched in the circulation of this patient population. LCN2 has emerged as a particularly intriguing candidate for mediating liver-muscle crosstalk. LCN2 has been described as a liver-to-brain mediator driving neuroinflammation and blood brain barrier dysfunction, demonstrating that liver-derived LCN2 can function as a bona fide endocrine signal in MASH [32]. Independent of liver disease, several models of muscle atrophy indicate that LCN2 can negatively modulate muscle health: global *Lcn2* knockout mice are protected from loss of muscle mass and strength decline during hindlimb suspension [33], and elevated LCN2 levels in mice with Duchenne muscular dystrophy contribute to muscle weakness, which is reversed upon *Lcn2* knockout [34]. Together, existing literature supports a role of LCN2 in both MASLD and muscle wasting, representing a promising link between the two metabolic disorders.

Similar to LCN2, osteopontin (OPN/*Spp1*) has been implicated in muscle wasting processes, particularly in dystrophic conditions like Duchenne muscular dystrophy and age-related sarcopenia, where it promotes fibrosis, skews macrophage polarization toward pro-inflammatory phenotypes, and impairs satellite cell regeneration, leading to reduced muscle mass and function [35,36]. Some studies report that *Spp1* ablation in mouse models of muscular dystrophy ameliorates pathology by decreasing TGF-β signaling and collagen expression, enhancing myotube hypertrophy via AKT/myostatin pathways [35,37], although this finding is not uniformly recapitulated [38]. In aged muscle, elevated OPN in macrophages inhibits myogenesis, with neutralization improving repair capacity [36]. OPN also correlates with chronic inflammation [39], an important driver of ubiquitin-proteasome activation in sarcopenia. Overall, despite its known role for both liver and muscle pathology, to date no direct connections between OPN, MASLD/MASH and sarcopenia have been established. In our models, OPN was particularly relevant in humans, with less effect in murine systems.

In conclusion, both OPN and LCN2 represent MASH- induced hepatokines in mice and humans with the potential to induce muscle wasting, representing targetable mechanisms for future interference with the MASH-sarcopenia bidirectional crosstalk.

## 4 Materials and methods

### 4.1 Patients

Human cohort studies were conducted at the University Hospital Aachen (RWTH Aachen), Germany. The study protocol was approved by the local ethics committee (EK 024/19) and conducted in accordance with the ethical standards laid down in the Declaration of Helsinki. Serum samples from male patients with advanced chronic liver disease (ACLD) with (n = 34; mean age 56.44 years) or without sarcopenia (n = 35; mean age 57.29 years) were analyzed. Sarcopenia was defined by computed tomography-derived skeletal muscle index (SMI) at the L3 level using a cutoff of <41.9 cm^2^/m^2^ according to previously published criteria [40]. Routine pre-interventional CT scans were used to determine SMI. SMI was measured using the 3D Slicer tool as previously described [41]. Grip strength was assessed with hand dynamometry with 1–3 measurements per side, and average values of the dominant hand were correlated with SMI. In the case that the dominant hand was not reported, values reported for the stronger hand were used. Samples were shipped on dry ice and stored at −70 °C until analysis.

### 4.2 Animal studies

Male C57BL/6N mice (Charles River Laboratories) were housed under specific pathogen-free conditions at 22 °C on a 12-h light/dark cycle with ad libitum access to food and water. Mice received standard chow (Provimi Kliba AG), a methionine and choline deficient diet containing 60% kcal fat (Research Diets A06071302i), or the GAN diet (Research Diets D09100310i). For the proteomics cohort including the MCD diet, control mice received the corresponding MCD control diet (10 kcal% fat; Research Diets A06071314, “Ctr”). All procedures were approved by the Government of Upper Bavaria (ROB-55.2-2532.Vet_02-17-49, ROB-55.2-2532.Vet_02-22-47, ROB-55.2-2532.Vet_02-22-64). Sample sizes are provided in the figure legends. At sacrifice, tissues were snap-frozen and stored at −70 °C unless otherwise indicated.

### 4.3 Body composition and muscle function

Body composition was determined by EchoMRI (Echo Medical Systems) according to the manufacturer's instructions. Four-limb grip strength was measured using a BIOSEB GT3 meter; three consecutive trials were performed per mouse and averaged. Four-limb hanging performance was evaluated using an inverted wire grid suspended ≥35 cm above soft bedding. Hang time was recorded across three trials with ≥2 min rest intervals and normalized to body weight.

### 4.4 AAV-mediated knockdown and adenoviral overexpression

For hepatocyte-specific knockdown, 10-12-week-old mice received 1 × 10^12^ AAV8 genomic copies per mouse (AAV8-Alb) via tail vein injection. shRNA constructs targeting *Lcn2*, *Lgals3*, or *Spp1*, as well as a non-targeting control (“scramble”), were custom-designed and produced by VectorBuilder; each vector encoded three distinct shRNA sequences and a GFP reporter. Organs were collected 6 weeks post-injection.

For hepatic overexpression, 10-12-week-old mice were injected via tail vein with 5 × 10^8^ viral particles per mouse of a replication-deficient adenovirus serotype 5 encoding *Lcn2* cDNA under control of a CMV promoter. The vector was generated in-house, and an empty adenoviral vector served as control. Viral titers were determined using the Adeno-X™ Rapid Titer Kit. Organs were collected 1.5 weeks post-injection.

### 4.5 Liver slices

Precision-cut liver slices were generated as previously described (de Graaf et al., 2010). Briefly, livers were excised, and 6-mm cores were prepared and maintained in ice-cold University of Wisconsin buffer. Slices (200–250 μm) were cut using an Alabama R&D Tissue Slicer in cooled Krebs–Henseleit buffer and transferred to Williams’ E medium supplemented with 2 mM glutamine and 50 μg/mL gentamicin. Slices were incubated at 37 °C under 5% CO_2_/95% O_2_ on an orbital shaker (60–90 rpm). After 1–3 h preincubation, medium was refreshed. After 16 h, supernatants and tissues were collected separately and stored at −70 °C.

### 4.6 Serum analysis

Tail vein blood (∼50 μL) was collected weekly during viral studies or at study termination in dietary experiments. Serum was isolated by centrifugation, snap-frozen, and stored at −70 °C. ALT and AST were quantified using a Beckman Coulter AU480 analyzer.

### 4.7 ELISAs

Serum LCN2, LGALS3, and OPN levels in mice and humans were quantified using DuoSet™ ELISA kits (R&D Systems; mouse: DY1857, DY1197, DY441; human: DY1757-05, DY1154, DY1433). Absorbance was measured at 450 nm (Varioskan™ LUX) with background correction at 540/570 nm. Concentrations were calculated from assay-specific standard curves. Serum dilutions were: LCN2 (mouse 1:5000; human 1:200), LGALS3 (mouse 1:4500; human 1:50), and OPN (mouse 1:5500; human 1:200).

### 4.8 Liver histology and MASH scoring

Formalin-fixed livers were paraffin-embedded, sectioned (3 μm), and stained with hematoxylin & eosin or Sirius Red (HistoCore SPECTRA ST, Leica) at the Pathology and Tissue Analytics Core Facility (Helmholtz Munich). Slides were digitized (AxioScan 7, Zeiss). MASH scoring was performed by a veterinary pathologist using established criteria for diet-induced murine liver disease (Finan et al., 2016).

### 4.9 Muscle immunofluorescence and lipid staining

Skeletal muscle cryosections (12 μm) were acetone-fixed, blocked (10% normal goat serum, 1% BSA), and incubated with antibodies against laminin (rabbit, 1:500; Sigma–Aldrich L9393) and myosin heavy chain isoforms BA-D5 (type I), SC-71 (type IIA), and BF–F3 (type IIB) (1:100; DSHB), followed by fluorophore-conjugated secondary antibodies (Jackson ImmunoResearch). Type IIX fibers were identified by absence of staining. Images were acquired using an Express2 slide scanner and analyzed with MuscleJ (ImageJ) for cross-sectional area and fiber-type distribution.

For neutral lipid visualization, adjacent sections were fixed in 4% paraformaldehyde, stained with BODIPY 493/503 (1 μg/mL) and DAPI (0.5 μg/mL), imaged by confocal microscopy (Olympus Fluoview), and BODIPY-positive particles quantified in ImageJ.

### 4.10 Primary hepatocytes

Mice were fed either chow or GAN diet for 27 weeks and primary hepatocytes were isolated according to a previously described protocol [42,43]. Following anesthesia with ketamine/xylazine, the abdominal cavity was opened to expose the liver, which was then perfused through the vena cava for 5–10 min with EGTA-containing HEPES/KH buffer, followed by perfusion with collagenase-containing HEPES/KH buffer for 10–15 min. Once digestion was visible, the liver was removed and placed in a petri dish containing suspension buffer, where it was gently agitated to facilitate cell release. The resulting cell suspension was filtered through a 100 nm pore mesh, and hepatocytes were washed twice by centrifuging 5 min at 50 g (4 °C). The hepatocyte pellet was subsequently dissolved in TRIzol for subsequent RNA isolation and quantitative PCR analysis.

### 4.11 Cell culture and treatments

Murine C2C12 myoblasts were cultured in DMEM (4.5 g/L glucose, l-glutamine, pyruvate) supplemented with 10% fetal bovine serum and 1% penicillin/streptomycin at 37 °C in 5% CO_2_. Cells were passaged at 50–60% confluence using 0.05% trypsin–EDTA and seeded at 5,000 cells/cm^2^. Myogenic differentiation was induced at confluence by switching to DMEM containing 2% horse serum for 3–7 days with medium renewal every other day. Cells were routinely tested for mycoplasma contamination by PCR. For cryopreservation, cells were frozen in growth medium containing 10% DMSO using controlled-rate cooling and stored in liquid nitrogen.

For stimulation experiments, differentiated myotubes (day 5–6) were treated for 48 h with precision-cut liver slice supernatant or recombinant mouse proteins: Lipocalin-2 (LCN2; R&D Systems, 1857-LC-050), Galectin-3 (LGALS3; R&D Systems, 1197-GA-050), or Osteopontin (OPN; R&D Systems, 441-OP-050) at 1–10 μg/mL. PCLS supernatant (16 h collection) was concentrated using 3 kDa molecular weight cut-off centrifugal filters prior to application.

### 4.12 Myotube diameter analysis

Differentiated C2C12 myotubes were fixed in 4% paraformaldehyde and stained with MitoTracker™ Green and DAPI. Images were acquired at 20 × magnification using a Nikon Eclipse Ts2-F microscope. Myotube diameter was quantified in ImageJ in a blinded manner by measuring three positions along each myotube, with ≥5 random fields analyzed per condition. A minimum of 500 myotubes were quantified per condition.

### 4.13 Human myotube culture

Primary human myoblasts were obtained from muscle biopsies as described previously [25]. In brief, muscle biopsies were taken from the lateral portion of the vastus lateralis of the quadriceps femoris after local anesthesia (2% Scandicaine; AstraZeneca, Germany) under sterile conditions using a fine-needle punch biopsy technique (Peter Pflugbeil GmBH, Germany). Biopsy donors (n = 10 individual donors age 29 ± 6 years, equal distribution between females and males) were participants of previous exercise intervention studies [[44], [45], [46], [47]]. All participants gave written informed consent and the study protocols were approved by the ethics committee of the University of Tübingen and in accordance with the declaration of Helsinki. Human satellite cells were released by collagenase digestion and seeded on 15-cm dishes coated with GelTrex™ (Thermo Fisher Scientific, Germany). After two rounds of proliferation in cloning medium (α-MEM:Ham's F-12 (1:1), 20% (v/v) FBS, 1% (v/v) chicken extract, 2 mM l-glutamine, 100 units/ml penicillin, 100 μg/ml streptomycin, 0.5 μg/ml amphotericin B), CD56- + myoblasts were enriched (>90%) using MACS microbeads and LS columns (Milteny Biotech, Germany), according to the manufacturer's protocol, with a 30-min incubation. They were then stored in the gaseous phase of liquid nitrogen. Cell culture surfaces were prepared with a non-gelling thin-layer GelTrex™ coating. Myoblasts (passage 3 after isolation, passage 1 after enrichment) were cultured in cloning media until 90% confluency. In 2D cultures, myotube differentiation was induced on day 0 and maintained for 7–10 days in differentiation media (α-MEM, 2 mM l-glutamine, 50 μM palmitate, 50 μM oleate (complexed to BSA with a final BSA concentration of 1.6 mg/ml in medium), 100 μM carnitine, 25 ng/ml IGF1 (human recombinant IGF1, I3769, Sigma–Aldrich, Germany)). Medium was changed three times per week and 48 h before harvest. For 3D skeletal muscle organoid generation, myoblasts at 90% confluency were trypsinated and cast within a collagen based extracellular matrix (ECM) containing 20% GelTrex, 10% TeloCol-6 (Advanced Biomatrix) and 500.000 myoblasts per organoid between a nylon frame (Cerex Advanced Fabrics) in a PDMS mold. Organoids were transferred to free floating culture in 6-wells with differentiation media after 24 h and differentiated for 7–10 days. On day 7 after full functional differentiation myotubes or 3D skeletal muscle organoids were treated with 1 μg/ml recombinant human LCN2, LGALS3 or 5 μg/ml OPN (R&D Systems) for 48h. To assess changes in diameter, 3 measurements of each organoid across its whole length were taken using microscopy images at 2.5x using a Axiovert 40C (Carl Zeiss Microscopy, Germany) and ImageJ and the mean of all three measurements was used. Controlled contraction was induced on day 9 of culture in 3D skeletal muscle organoids at 0.5 Hz 4 ms 15 V using C-Pace EP (Ionoptix, USA). Contraction was analyzed using a motion tracking algorithm (open heart ware) [3] on 15 s captured videos during EPS using a Axiovert 40C (Carl Zeiss Microscopy, Germany) with Flexacam C3 Camera (Leica, Germany) to calculate maximum contraction speed (delta v(μm/s)) and contraction distance (area under the curve (AUC)). Mycoplasma free culture conditions and cells are regularly controlled for using the MycoAlert Mycoplasma Detection Kit (Lonza, Switzerland).

### 4.14 Mitochondrial respiration C2C12 and human myotubes

Oxygen consumption rate (OCR) was measured using a Seahorse XFe96 or 24 Extracellular Flux Analyzer (Agilent Technologies). C2C12 myotubes were differentiated in XF96 and human myotubes in XF24 microplates and analyzed after 48 h of treatment (≥3–5 technical replicates per condition). Prior to measurement, cells were incubated for 1 h in Seahorse XF Base Medium supplemented with 10 mM glucose, 1 mM pyruvate, and 2 mM l-glutamine (pH 7.4) at 37 °C in a non-CO_2_ incubator.

A mitochondrial stress test was performed by sequential injection of oligomycin (1 μM C2C12 and human myotubes), FCCP (4 μM C2C12, 2 μM human myotubes), and antimycin A/rotenone (0.75 μM each C2C12, 0.5 μM human myotubes). OCR was recorded in repeated mix'-wait-measure cycles. Basal respiration, ATP-linked respiration, proton leak, maximal respiration, spare respiratory capacity, and non-mitochondrial respiration were calculated according to standard definitions.

### 4.15 RNA isolation and quantitative PCR

Total RNA from C2C12 cells and frozen tissues was extracted using TRIzol reagent (Invitrogen) followed by phase separation and alcohol precipitation. Cell-derived RNA was additionally purified using silica membrane spin columns. RNA concentration and purity were assessed by NanoDrop spectrophotometry. After DNase I treatment (Life Technologies), 100–1000 ng RNA was reverse-transcribed using the High-Capacity cDNA Reverse Transcription Kit (Life Technologies).

Quantitative PCR was performed using Takyon™ Low Rox Probe MasterMix and TaqMan assays on a QuantStudio™ 6/7 Flex system (Applied Biosystems). The following mouse probes were used: *Fbxo32* (Mm00499523_m1), *Lcn2* (Mm01324470_m1), *Lgals3* (Mm00802901_m1), *Spp1/Opn* (Mm00436767_m1), *Trim63* (Mm01185221_m1), *Hprt* (Mm03024075_m1), *Rer1* (Mm00471276_m1), and Tbp (Mm01277042_m1). Gene expression was calculated using the ΔCt method. *Hprt* was used for normalization of liver samples, and the geometric mean of *Hprt*, *Tbp*, and *Rer1* for muscle samples.

### 4.16 Protein expression analysis

Cells and tissues were lysed in RIPA buffer containing protease and phosphatase inhibitors. Proteins were quantified using the Pierce™ BCA assay and separated by SDS PAGE (Novex™ Tris-Glycine gels). Proteins were transferred to nitrocellulose membranes, probed with primary and HRP-conjugated secondary antibodies (Supplemental Table 4), and visualized by chemiluminescence (ChemiDoc™, Bio-Rad). Band intensities were quantified using Image Lab software (Bio-Rad) and normalized to Vinculin or GAPDH as indicated.

### 4.17 Proteomics

Proteomic analyses were performed at the Core Facility of Metabolomics and Proteomics (Helmholtz Munich). Muscle samples were prepared and MS measurement was performed as described [48]. In short, samples were digested in-solution overnight and peptides were stored at −20 °C after cleanup. LC-MSMS analysis was performed in data-dependent acquisition (DDA) mode. MS data were acquired on a Q-Exactive HF-X mass spectrometer (Thermo Scientific) each online coupled to a nano-RSLC (Ultimate 3000 RSLC; Dionex). Data analysis for whole proteome was performed using MaxQuant 2.0.3.1. and Perseus 1.6.14.0.

For secretome proteomics, equal protein amounts of samples were subjected to tryptic digestion applying a modified filter-aided sample preparation (FASP) procedure as described in Meul et al. [49]. MS measurement was performed as described for muscle samples. Data analysis was performed using Proteome Discoverer 2.5 software (Thermo Fisher Scientific) using the Sequest HT search engine against the SwissProt mouse database (release 2020_02, 17,061 sequences).

### 4.18 Metabolite extraction and GC–MS analysis

For isotope tracing experiments, C2C12 myotubes were incubated with supernatant (SN) collected from live liver slice cultures in the presence of U–^13^C_6_-glucose. Metabolites were subsequently extracted as previously described. [50]. Briefly, cells were washed with 0.9% NaCl and quenched with ice-cold methanol and ddH_2_O containing 1 μg/mL D6-glutaric acid as an internal standard. Cells were scraped, and extracts were transferred to tubes containing ice-cold chloroform. Samples were vortexed at 1400 rpm for 20 min at 4 °C and centrifuged at 17,000 g for 5 min at 4 °C to achieve phase separation. The upper polar phase (300 μL) was transferred to GC glass vials with micro-inserts and dried under vacuum at 4 °C.

Dried extracts were derivatized by methoxylamine hydrochloride (20 mg/mL in pyridine) followed by N-tert-butyldimethylsilyl-N-methyltrifluoroacetamide (MTBSTFA). Metabolite levels and isotopic enrichment were analyzed using an Agilent 7890B Gas Chromatograph coupled to an Agilent 5977 Mass Selective Detector, equipped with a DB-35 ms capillary column (30 m). Samples (1 μL) were injected in splitless mode with helium as carrier gas. Data were acquired in selected ion monitoring mode. Chromatograms were processed to determine relative metabolite abundances and mass isotopomer distributions corrected for natural occurring isotopes using MetaboliteDetector software as previously described [51,52].

### 4.19 Statistical analysis

Data are presented as mean ± SEM. Statistical analyses were performed using GraphPad Prism (v10.5.0). Normality was assessed using D'Agostino-Pearson and Shapiro–Wilk tests. Two-group comparisons were analyzed by two-tailed Student's t-test or Mann–Whitney test, as appropriate. Two-way ANOVA with Tukey's or Bonferroni post hoc testing was applied for multiple comparisons. P < 0.05 was considered significant.

## 5 Limitation of the study

All data shown was generated using male mice, and we explored exclusively male patients. Future studies will investigate sex differences in the bidirectional MASLD-sarcopenia crosstalk.

## CRediT authorship contribution statement

**Amy R. Fumo:** Writing – review & editing, Visualization, Methodology, Investigation, Formal analysis, Data curation, Conceptualization. **Simon I. Dreher:** Writing – review & editing, Methodology. **Pauline Morigny:** Writing – review & editing, Methodology. **Honglei Ji:** Writing – review & editing, Methodology. **Raul Terron Exposito:** Writing – review & editing, Methodology. **Tuna F. Samanci:** Writing – review & editing, Methodology. **Tushar More:** Writing – review & editing, Methodology. **Lara Ruoff:** Writing – review & editing, Methodology. **Joël J. Tissink:** Writing – review & editing, Methodology. **Carmen Paredes Yubero:** Writing – review & editing, Methodology. **Ana Jimena Alfaro:** Writing – review & editing, Methodology. **Christine von Törne:** Writing – review & editing, Methodology. **Stefanie Hauck:** Writing – review & editing, Methodology. **Sophie H.A. Nusser:** Writing – review & editing, Methodology. **Zoltan Czigany:** Writing – review & editing, Methodology. **Kenneth A. Dyar:** Writing – review & editing, Methodology. **Stephan Herzig:** Writing – review & editing, Investigation. **Mauricio Berriel Diaz:** Writing – review & editing, Investigation. **Anja Zeigerer:** Writing – review & editing, Investigation. **Karsten Hiller:** Writing – review & editing, Investigation. **Theresa H. Wirtz:** Writing – review & editing, Investigation. **Cora Weigert:** Writing – review & editing, Investigation. **Maria Rohm:** Writing – original draft, Supervision, Funding acquisition, Conceptualization.

## Declaration of generative AI and AI-assisted technologies in the manuscript preparation process

During the preparation of this work A.R.F. and M.R. used Perplexity to identify relevant literature and improve clarity and flow of the text. After using this tool/service, the authors reviewed and edited the content and take full responsibility for the content of the published article.

## Declaration of competing interest

The authors declare the following financial interests/personal relationships which may be considered as potential competing interests: The project was partly funded through a research grant through the Helmholtz Munich - Novo Nordisk strategic alliance to M.R. All other authors declare no conflict of interest.

## Data Availability

Data available online.
