# Supplementary figures and images for: Hepatokines lipocalin 2 and osteopontin drive muscle atrophy in MASH
Source: Mol Metab. 2026 Jun 10;110:102391. doi: 10.1016/j.molmet.2026.102391 (PMC13320412; doi:10.1016/j.molmet.2026.102391)

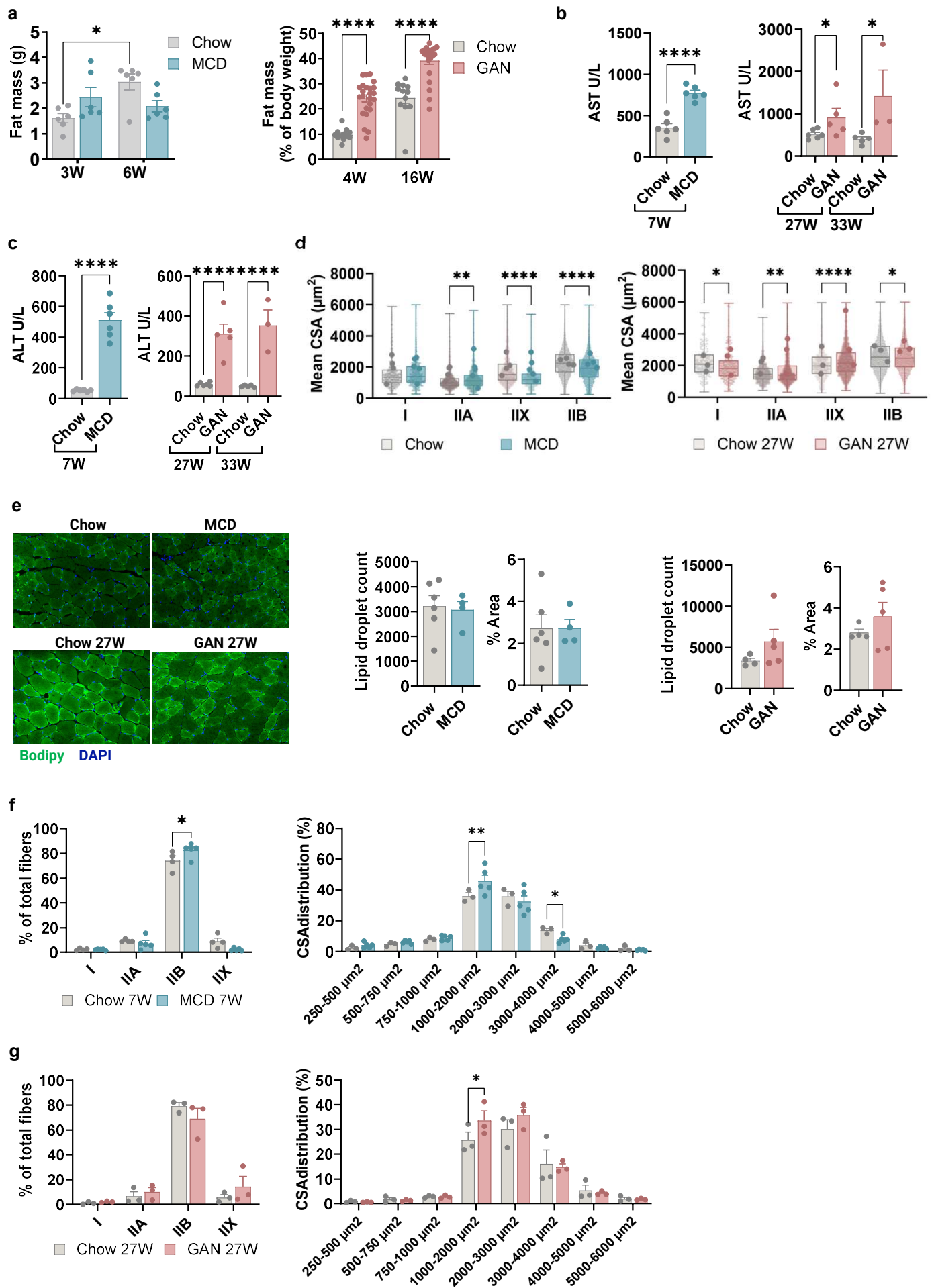

**a**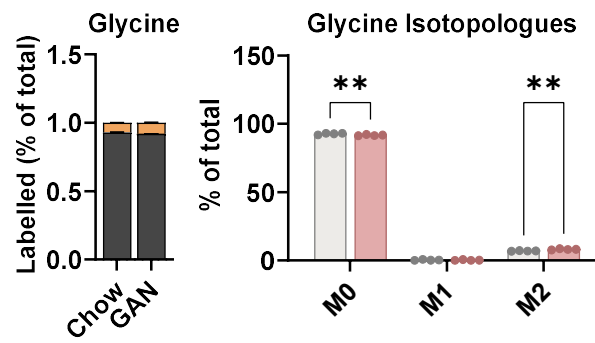**b**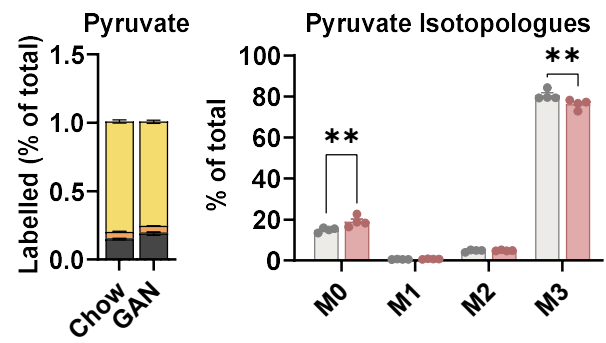**c**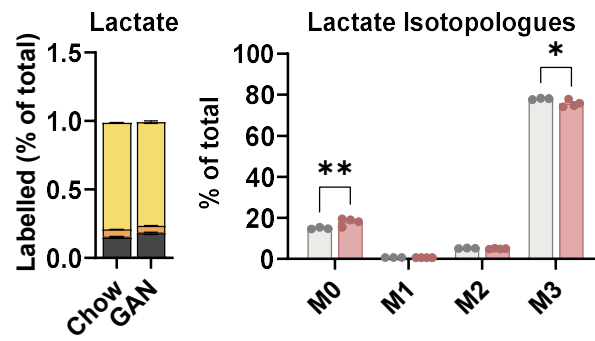

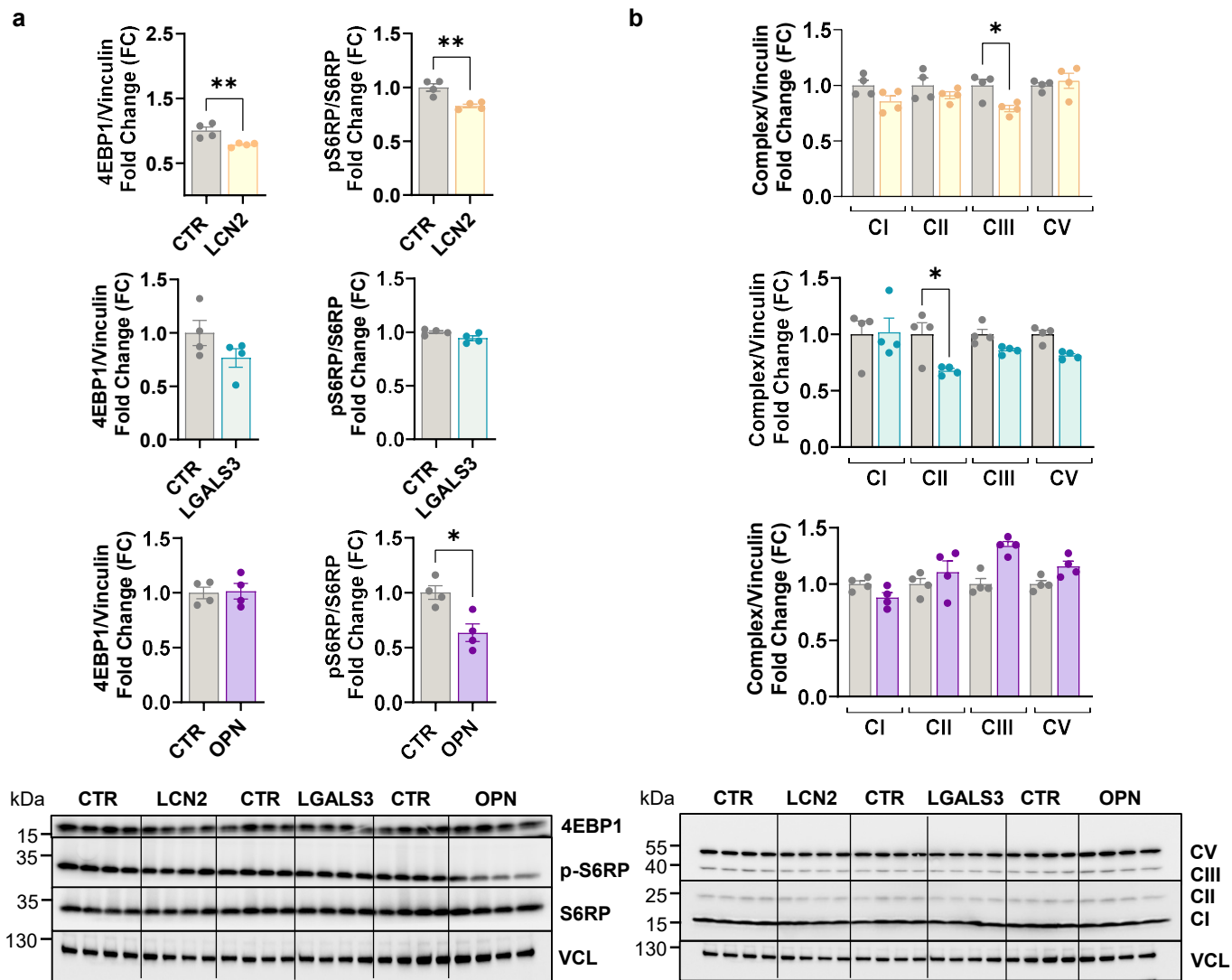

**a**

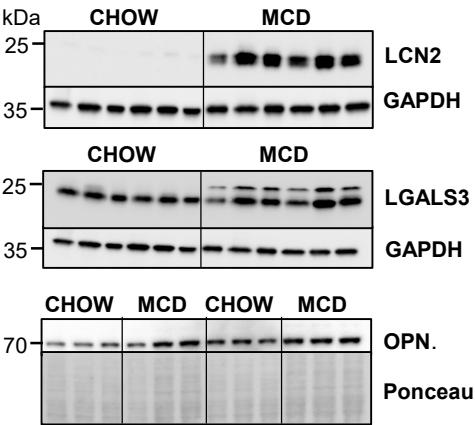

**b**

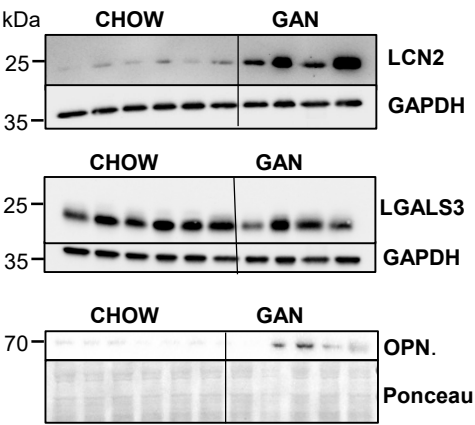

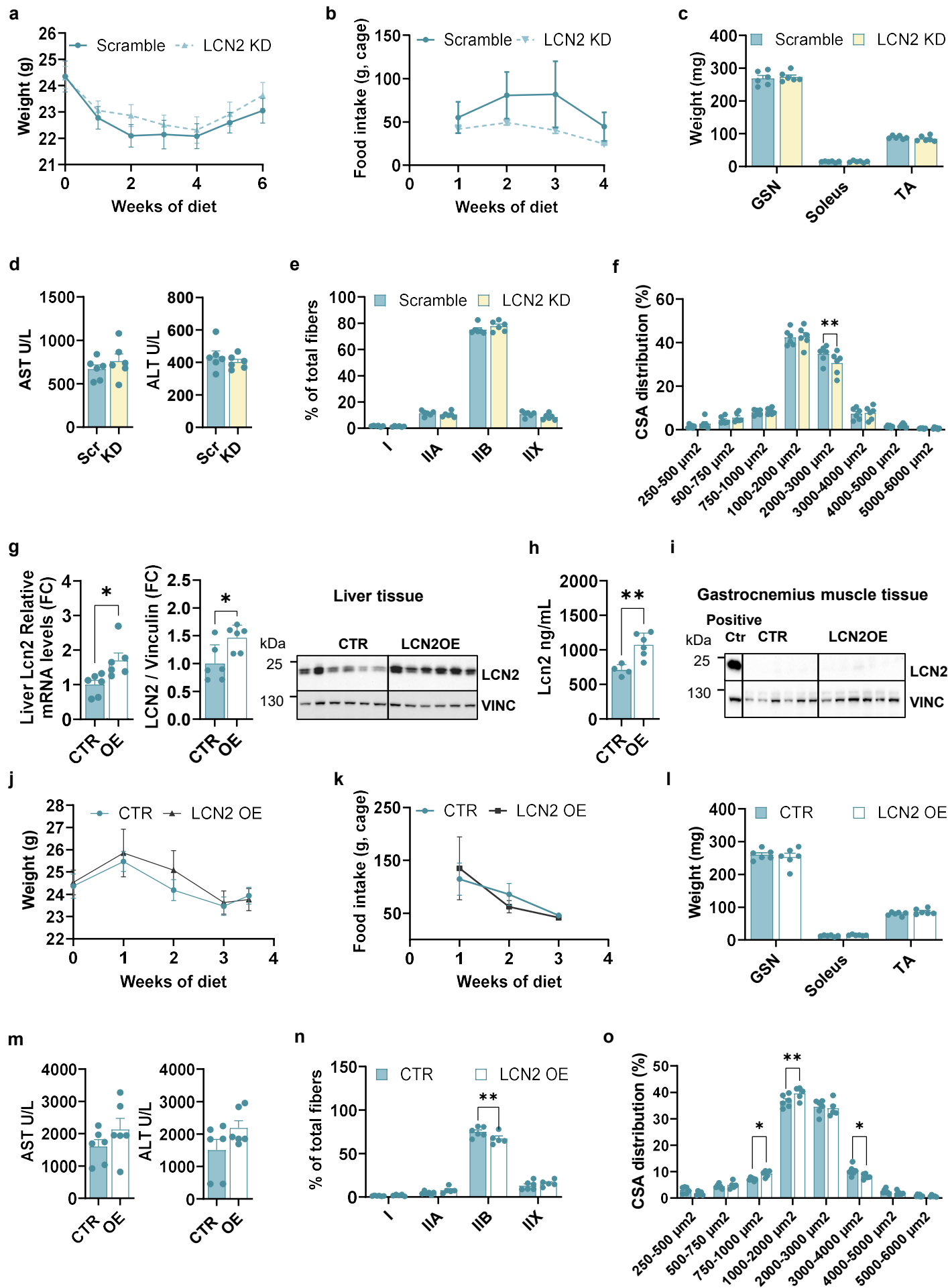

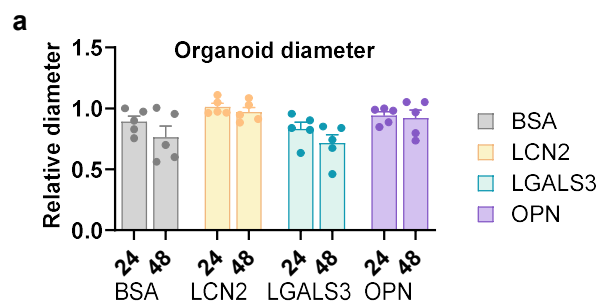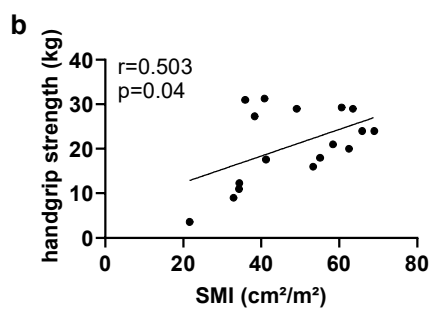

Supplement: Multimedia component 4 [file mmc4.pdf]
