# Supplementary material for: Hepatokines lipocalin 2 and osteopontin drive muscle atrophy in MASH
Source: Mol Metab. 2026 Jun 10;110:102391. doi: 10.1016/j.molmet.2026.102391 (PMC13320412; doi:10.1016/j.molmet.2026.102391)
Supplement: Multimedia component 5 [file mmc5.docx]

**Supplemental Figure Legends**

**Supplemental Fig. 1. MCD and GAN models exhibit liver injury and distinct gastrocnemius fiber adaptations. (a)** Fat mass measured by EchoMRI in MCD and GAN cohorts compared to respective chow controls. MCD/chow 3W & 6W: n = 6 per group; GAN/chow 4W & 16W: chow n = 12, GAN n = 24 (4W) and n = 22 (16W). **(b)** Serum AST levels at study endpoint in MCD and GAN cohorts. MCD/chow n = 6 per group; GAN 27W n = 5, chow 27W n = 6; GAN 33W n = 3, chow 33W n = 5. **(c)** Serum ALT levels at study endpoint (n as in b). **(d)** Fiber type-specific cross-sectional area (CSA) in gastrocnemius muscle from MCD and GAN mice. **(e)** Lipid droplet number and lipid droplet area (%) in gastrocnemius muscle, assessed by BODIPY staining. Representative BODIPY/DAPI images from MCD, GAN (27W), and respective chow controls are shown. MCD n = 4, chow n = 6; GAN 27W n = 5, chow 27W n = 4. **(f)** Fiber type and cross sectional area (CSA) distribution in gastrocnemius muscle of MCD and Chow mice. Values are expressed as percentage of total fibers. Chow n = 3, MCD n = 5. **(g)** Corresponding fiber type and cross-sectional area (CSA) distribution analysis in gastrocnemius muscle of GAN and Chow mice after 27W of diet. Chow n = 3, GAN n = 4. Data are presented as mean ± SEM. Statistical analyses were performed using two-tailed Student’s t-test or one-/two-way ANOVA with appropriate post hoc testing as indicated. *P < 0.05, **P < 0.01, ***P < 0.001, ****P < 0.0001.

**Supplemental Fig. 2.** **Glycine and glycolytic labeling remain largely preserved in myotubes exposed to GAN-derived liver slice supernatant.** C2C12 myotubes were treated for 48 h with supernatant derived from liver slices from 27 week old chow- or GAN-fed mice and subsequently incubated with [U-¹³C]glucose for stable isotope tracing. **(a)** Fractional labeling (% of total metabolite pool) and isotopologue distribution of glycine, **(b)** pyruvate, and **(c)** lactate. Isotopologue distributions are shown as the relative abundance (% of total) of M0-M3 isotopologues. Data represent mean ± SEM (n = 4). Statistical analyses were performed using two-way ANOVA with Bonferroni post hoc test. *P < 0.05, **P < 0.01**.**

**Supplemental Fig. 3. Recombinant proteins alter electron transport chain composition and mTOR signaling in C2C12 myotubes. (a)** 4E-BP1, Phosphorylated S6 ribosomal protein (pS6RP) and total S6RP in C2C12 myotubes treated with recombinant LCN2, LGALS3, or OPN, with densitometric quantification (n=4). **(b)** Western blots of oxidative phosphorylation (OXPHOS) complexes in C2C12 myotubes treated with recombinant LCN2, LGALS3, or OPN, with corresponding densitometric quantification (n = 4). Data are presented as mean ± SEM. Statistical analyses were performed using two-tailed Student’s t-test or one-way ANOVA with Šídák's multiple comparison post hoc test. *P < 0.05, **P < 0.01.

**Supplemental Fig. 4. Hepatic expression of LCN2, LGALS3, OPN in MCD and GAN models**. Western blots showing hepatic protein expression of LCN2, LGALS3, and OPN in MCD **(a)** and 27 week fed GAN models **(b)**.

**Supplemental Fig. 5. Hepatocyte-specific LCN2 knockdown or overexpression does not alter body weight, muscle mass, or food intake**. **(a)** Body weight of MCD-fed mice following AAV-mediated *Lcn2* knockdown (KD) compared to scramble controls (n = 6 per group). **(b)** Food intake per cage (3 mice per cage) during *Lcn2* KD experiments (n = 4 cages per group) **(c)** Muscle weights at experimental endpoint (gastrocnemius, soleus, tibialis anterior) in MCD-fed mice following *Lcn2* knockdown (n = 6 per group). **(d)** Serum AST and ALT levels (n = 6 per group). **(e)** Percentage of total muscle fibers per fiber type following *Lcn2* knockdown. **(f)** Cross-sectional area (CSA) distribution of muscle fibers following Lcn2 knockdown. Data are shown as percentage of total fibers per CSA bin. **(g)** Gene expression and protein levels of *Lnc2* in livers of MCD-fed mice following adenovirus mediated overexpression (OE) of *Lcn2* **(h).** Serum LCN2 levels measured by ELISA (n = 6 per group). **(i)** Western blot of LCN2 in GSN muscle following adenovirus-mediated *Lcn2* overexpression, confirming absence of ectopic muscle overexpression. **(j)** Body weight progression following adenovirus mediated *Lcn2* overexpression (OE) compared to empty vector controls (n = 6 per group). **(k)** Food intake per cage during *Lcn2* OE (n = 4 cages per group, 3 mice per cage). **(l)** Muscle weights at experimental endpoint (gastrocnemius, soleus, tibialis anterior) following *Lcn2* OE (n = 6 per group). **(m)** Serum AST and ALT levels (n = 6 per group). **(n)** Percentage of total muscle fibers per fiber type following *Lcn2* OE. **(o)** Cross-sectional area (CSA) distribution of muscle fibers following Lcn2 overexpression. Data are shown as percentage of total fibers per CSA bin. Data are presented as mean ± SEM. Statistical analyses were performed using two-tailed Student’s t-test or two-way ANOVA with Šídák's multiple comparison post hoc test or uncorrected Fisher’s LSD as appropriate. *P < 0.05, **P < 0.01.

**Supplemental Fig. 6. Unchanged muscle organoid size upon treatment with recombinant protein. (a)** 3D human skeletal muscle organoids were treated with 1µg/mL of recombinant human LCN2, LGALS3 or 5µg/mL OPN for 24 and 48 h. Whole organoid diameter was measured in microscopic images as mean of 3 points across the whole length of the organoid via ImageJ. Organoid diameter is shown as fold change relative to baseline (t₀). Data are presented as mean ± SEM. Statistical analyses were performed using two-way ANOVA with Šídák's multiple comparison post hoc test. **(b)** Correlation between skeletal muscle index (SMI) and handgrip strength in patients with ACLD (n=16). Statistical significance and correlation coefficient were determined using Pearson correlation analysis.

**Supplemental Table 1. MCD mice muscle proteomics.** Proteomic analysis of gastrocnemius (GSN) muscle of CTR and MCD-fed mice (7W, n = 4 per group, matching Figure 2).

**Supplemental Table 2. GAN mice muscle proteomics.** Proteomic analysis of gastrocnemius (GSN) muscle of chow- and GAN-fed mice (27W, n = 4 per group, matching Figure 2).

**Supplemental Table 3. Proteomics analysis of supernatant of live liver slices.** Secretome proteomics of liver-derived supernatant from chow (n = 5) and MCD (n = 6, 7 weeks diet) mice (matching Figure 4).
