# Supplementary material for: Hepatokines lipocalin 2 and osteopontin drive muscle atrophy in MASH
Source: Mol Metab. 2026 Jun 10;110:102391. doi: 10.1016/j.molmet.2026.102391 (PMC13320412; doi:10.1016/j.molmet.2026.102391)
Supplement: Multimedia component 6 [file mmc6.docx]

**Supplemental Table 4. Antibodies used in this study**

| **Target** | **Host** | **Supplier** | **Catalog #** | **Dilution** | **Application** |
| --- | --- | --- | --- | --- | --- |
| Vinculin | Rabbit | Abcam | ab129002 | 1:10,000 | WB (loading control muscle) |
| GAPDH | Rabbit | Cell Signaling Technology | 2118 | 1:1,000 | WB (loading control liver) |
| S6RP (5G10) | Rabbit | Cell Signaling Technology | 2217S | 1:1,000 | WB |
| Phospho-S6RP (Ser235/236) | Rabbit | Cell Signaling Technology | 2211S | 1:1,000 | WB |
| Lcn2 | Goat | Bio-Techne | AF1857 | 1:1,000 | WB |
| Galectin-3 (B2C10) | Mouse | Santa Cruz Biotechnology | sc-32790 | 1:500 | WB |
| Total OXPHOS Antibody Cocktail | Mouse | Abcam | ab110413 | 1:1,000 | WB |
| Osteopontin (Opn) | Mouse | Proteintech | 83341-1-RR | 1:2,000 | WB |
| GFP | Rabbit | Proteintech | 50430-2-AP | 1:10,000 | WB |
| Anti-mouse IgG-HRP | Goat | Cell Signaling Technology | 2368 | 1:5,000 | WB secondary |
| Anti-rabbit IgG-HRP | Goat | Cell Signaling Technology | 60433 | 1:5,000 | WB secondary |
| Anti-goat IgG-HRP | Rabbit | Bio-Techne | HAF017 | 1:5,000 | WB secondary |
| Anti-mouse IgG1 CF™488A | Goat | Sigma-Aldrich | SAB4600238 | 1:100 | IF secondary |
| Anti-mouse IgG2b CF™405S | Goat | Sigma-Aldrich | SAB4600477 | 1:100 | IF secondary |
| Anti-rabbit IgG CF™647 | Goat | Sigma-Aldrich | SAB4600185 | 1:500 | IF secondary |
| Anti-mouse IgM DyLight™ 594 | Goat | Invitrogen | SA5-10152 | 1:100 | IF secondary |
